# Early Hyperdynamic Sepsis Alters Coronary Blood Flow Regulation in Porcine Fecal Peritonitis

**DOI:** 10.3389/fphys.2021.754570

**Published:** 2021-12-03

**Authors:** Céline Boudart, Fuhong Su, Lorenzo Pitisci, Arnaud Dhoine, Olivier Duranteau, Pascale Jespers, Antoine Herpain, Rebecca Vanderpool, Serge Brimioulle, Jacques Creteur, Robert Naeije, Luc Van Obbergh, Laurence Dewachter

**Affiliations:** ^1^Department of Anesthesiology, Erasme University Hospital, Université Libre de Bruxelles, Brussels, Belgium; ^2^Laboratory of Physiology and Pharmacology, Faculty of Medicine, Université Libre de Bruxelles, Brussels, Belgium; ^3^Department of Intensive Care, Erasme University Hospital, Université Libre de Bruxelles, Brussels, Belgium; ^4^Division of Translational and Regenerative Medicine, Department of Medicine, The University of Arizona College of Medicine, Tucson, AZ, United States

**Keywords:** coronary blood flow, autoregulation, sepsis, metabolic regulation, endothelial function, microcirculation

## Abstract

**Background:** Sepsis is a common condition known to impair blood flow regulation and microcirculation, which can ultimately lead to organ dysfunction but such contribution of the coronary circulation remains to be clarified. We investigated coronary blood flow regulatory mechanisms, including autoregulation, metabolic regulation, and endothelial vasodilatory response, in an experimental porcine model of early hyperdynamic sepsis.

**Methods:** Fourteen pigs were randomized to sham (*n* = 7) or fecal peritonitis-induced sepsis (*n* = 7) procedures. At baseline, 6 and 12 h after peritonitis induction, the animals underwent general and coronary hemodynamic evaluation, including determination of autoregulatory breakpoint pressure and adenosine-induced maximal coronary vasodilation for coronary flow reserve and hyperemic microvascular resistance calculation. Endothelial-derived vasodilatory response was assessed both *in vivo* and *ex vivo* using bradykinin. Coronary arteries were sampled for pathobiological evaluation.

**Results:** Sepsis resulted in a right shift of the autoregulatory breakpoint pressure, decreased coronary blood flow reserve and increased hyperemic microvascular resistance from the 6th h after peritonitis induction. *In vivo* and *ex vivo* endothelial vasomotor function was preserved. Sepsis increased coronary arteries expressions of nitric oxide synthases, prostaglandin I_2_ receptor, and prostaglandin F_2α_ receptor.

**Conclusion:** Autoregulation and metabolic blood flow regulation were both impaired in the coronary circulation during experimental hyperdynamic sepsis, although endothelial vasodilatory response was preserved.

## Introduction

Sepsis is a condition of life-threatening organ dysfunction caused by an inappropriate host response to infection (Singer et al., 2016). During hyperdynamic sepsis with sustained or even supranormal cardiac output, regional manifestations of hypoperfusion (lactic acidosis, decreased urine output, or altered mental state) may persist and ultimately result in organ dysfunction. Indeed, brain circulation has shown sepsis-related microcirculation (Taccone et al., 2010) and autoregulation impairment (Schramm et al., 2012; Crippa et al., 2018; Ferlini et al., 2020), associated with brain dysfunction (Schramm et al., 2012; Crippa et al., 2018) and neurovascular uncoupling (Ferlini et al., 2020). Such disturbances of flow regulation in the coronary circulation and their potential implication on myocardial function still need to be investigated.

To preserve adequate supply of oxygen and metabolites to myocardial cells, coronary blood flow is tightly regulated, combining neuro-endocrine, paracrine and mechano-sensitive pathways. These regulatory mechanisms maintain the coronary blood flow constant, countering potential variations in coronary perfusion pressure (autoregulation) (Mosher et al., 1964; Canty and Jr, 1988), and provide blood supply perfectly adapted to the metabolic myocardial requirements, a mechanism called metabolic regulation (Feigl et al., 1990; Tune et al., 2004). In physiological conditions, coronary blood flow may triple when required; this maximal increase represents the coronary flow reserve (Kern et al., 2006). Microcirculation is the main site of these regulatory mechanisms and contributes over 90% of coronary vascular resistance. Coronary blood flow is controlled by both systemic and locally released molecules acting directly on local vascular smooth muscle. In response to physical or metabolic stimuli acting on vessel walls, the endothelium releases vasoactive molecules, including eicosanoids (prostaglandins and thromboxane), nitric oxide (NO), endothelium-derived hyperpolarizing factor (EDHF), and endothelin-1, to modulate coronary vascular tone.

Human coronary blood flow has been studied extensively in healthy subjects and in patients with coronary artery disease. However, the role and character of hemodynamic changes in the coronary circulation during sepsis, as described in other vascular beds, remain incompletely understood. In this context, we hypothesized that sepsis-related microvascular dysfunction observed in other vascular beds may also affect the coronary circulation and thereby alter its regulatory mechanisms. The aim of the present physiological study was to assess coronary regulatory mechanisms in a porcine model of hyperdynamic sepsis induced by fecal peritonitis.

## Materials and Methods

Fourteen 4-month old domestic pigs (male:female; 7:7) weighting 38 ± 4 kg were included in this study, which was approved by the Institutional Ethics Committee on Animal Welfare from the Faculty of Medicine from the *Université Libre de Bruxelles* (Brussels, Belgium; Acceptation Number 624N). All animals received care and were handled in agreement with the “Guide for Care and Use of Laboratory Animals” published by the U.S. National Institutes of Health (NIH Publication eighth edition, update 2011). The animals were fasted for 12 h with free access to water, before the start of the experiment.

### Animal Equipment

The animals were premedicated, anesthetized, mechanically ventilated, and equipped, as previously reported (Boudart et al., 2020). Briefly, a five-lead electrocardiogram was placed to monitor heart rate (HR). Two 5-F high-fidelity pressure catheters (Transonic^®^ System Inc., New York, United States) were positioned under fluoroscopy guidance in the aortic arch and in the left ventricle to measure aortic and left ventricular pressures, respectively. A balloon-tipped thermodilution pulmonary artery catheter (Swan-Ganz^®^, Edwards Life Sciences, Irvine, United States) was placed in the pulmonary artery, under the monitoring of pressure wave contours, to measure pulmonary arterial pressure and cardiac output (CO) (Vigilance^®^, Edwards Life Sciences, Irvine, United States). A 0.014 inch pressure-flow sensor-tipped guidewire (ComboWire^®^, Volcano Corporation, San Diego, CA, United States) was placed in the mid-portion of the left anterior descending coronary artery to perform intracoronary hemodynamic measurements. An intracoronary dilatation balloon (Trek^®^, Abbott Vascular, Santa Clara, United States) was placed over the guidewire in the proximal part of the left anterior descending coronary artery. Two multiperforated silicon drains were surgically placed into paracolic gutters for sepsis induction. Arterial blood sampling was performed every hour for blood gas analysis. Normoxia (PaO_2_ 90–100 mm Hg), normocapnia (PaCO_2_ 35–45 mm Hg), normothermia (37.5–38.5°C), and normoglycemia (80–120 mg/ml) were permanently adjusted during the whole protocol.

### Experimental Protocol and Tissue Sampling

Pigs were randomly assigned to sham (*n* = 7) or sepsis procedures (*n* = 7), with equal distribution of gender in both groups (the ratio of male and female pigs was 3:4 and 4:3 in the sham and the sepsis groups, respectively). After a 2 h period, to ensure post-equipment stabilization and baseline measurements, fecal peritonitis was induced by inoculation of autologous feces suspension (3 mg/kg autologous feces suspended in 250 ml saline solution and cultured at room temperature during animal preparation and stabilization time) into the abdominal cavity through two multiperforated silicon drains. These were kept clamped for 5 h and thereafter released before the 6 h sepsis measurement. In both groups, balanced crystalloid solution (PlasmaLyte^®^, Baxter SA, Lessine, Belgium) was administered (at 10 ml/kg/h during instrumentation and surgical procedure and at 7 ml/kg/h during the rest of the protocol) as maintenance fluid administration. In the sepsis group, a colloid solution (Voluven^®^, Fresenius Kabi, Schelle, Belgium) was administered according to sepsis development (3–15 ml/kg/h) to maintain mean arterial pressure (MAP) close to its baseline value and according to CO responsiveness.

Data were acquired at baseline, 6 and 12 h after sepsis induction by fecal peritonitis. General and coronary hemodynamic measurements were recorded and analyzed using Notocord-hem Evolution^®^ Software (NOTOCORD Systems SAS, Le Pecq, France).

At the end of the protocol, the animals were euthanized, and the heart was immediately explanted. Epicardial coronary artery vessels were carefully dissected and cleaned of adhesive fat and connective tissue, and artery sections of 5 mm length were immediately harvested for *ex vivo* vasoreactivity experiments. Coronary samples were also collected and snap-frozen in liquid nitrogen and stored at −80°C for pathobiological evaluation.

### General Hemodynamic Measurements

Each data set included measurements of HR, MAP, left ventricular end-diastolic pressure (LVEDP), mean pulmonary arterial pressure (MPAP), and CO. Left and right ventriculo-arterial coupling were also evaluated at baseline, 6 and 12 h measurements. End-systolic elastance (Ees) of left and right ventricle and arterial elastance (Ea) of the aorta and pulmonary artery were determined from the analysis of the ventricular pressure curve using the single beat method as previously reported (Sunagawa et al., 1980; Brimioulle et al., 2003; Singh et al., 2019). Briefly, this method relies on a maximum pressure (Pmax) calculation from a sinusoidal extrapolation of early and late portions of a ventricle pressure curve. Ees and Ea were calculated as follows:


$$E\,e\,s=\frac{P\,m\,a\,x-E\,S\,P}{S\,V},E\,a=\frac{E\,S\,P}{S\,V}$$

where ESP represents the end-systolic pressure measured by a high-fidelity pressure catheter respectively placed in the left or right ventricle, and SV is the stroke volume. The coupling between the ventricular (left or right ventricular) function to the arterial (aorta or pulmonary artery) circulation was estimated by the Ees/Ea ratio.

### Coronary Hemodynamic Measurements

Coronary pressure and flow velocities were subsequently recorded in resting conditions, during hyperemia (maximal vasodilation), achieved first with endothelium-independent vasodilator adenosine (3 mL bolus of 90 μg; Adenocor^®^, Sanofi, Diegem, Belgium) and then with endothelium-dependent vasodilator bradykinin (3 ml bolus of 10^–6^ mol/L; Sigma–Aldrich Chemicals, Overijse, Belgium). Resting flow was calculated as the mean of five successive beats at rest. Hyperemic flow was calculated as the mean of three successive beats at maximal vasodilation. Coronary flow reserve was defined as the ratio between hyperemic and resting flows. Hyperemic coronary microvascular resistance was defined as the ratio between coronary pressure and hyperemic flow during maximal vasodilation (Kern et al., 2006). For both the vasodilatory agents (adenosine and bradykinin), three representative measurements were averaged. Sufficient time was respected between each measurement to ensure a return to the prior resting conditions.

Coronary pressure–flow relationship was obtained by progressive and careful inflation of the intra-coronary balloon until abrupt fall of coronary blood flow, to determine the lower pressure limit of autoregulation, as previously described (Boudart et al., 2020). Briefly, paired sets of linear regressions and their coefficient of determination R-squared (R^2^) were calculated, in pressure steps of 5 mm Hg, starting at the highest and lowest pressures. The autoregulatory breakpoint pressure was determined as the intersection of the two best-fitted regression lines.

### *Ex vivo* Evaluation of Coronary Endothelial-Dependent Vascular Reactivity

Dissected epicardial coronary artery rings of 3 mm length and 2.5 mm diameter were mounted on stainless steel hooks in 5 ml-organ chambers filled with Krebs-Henseleit solution (118.1 mmol/L NaCl; 4.7 mmol/L KCl; 1.2 mmol/L MgSO_4_; 1.2 mmol/L KH_2_PO_4_; 2.5 mmol/L CaCl_2_; 25 mmol/L NaHCO_3_; 5.1 mmol/L glucose) bubbled with mixed 95% O_2_ and 5% CO_2_ and maintained at 37°C. During dissection, care was taken to protect the endothelial lining in the coronary artery rings. One of the steel hooks was anchored in the chamber, and the other was connected to a force transducer for continuous recording of isometric tension with a force-displacement transducer (IOSlab^®^ version 4.35, EMKA Technologies, Paris). Each ring was progressively submitted to a resting tension of 2 g and allowed to equilibrate for a period of 60 min. During this time, Krebs solution was fully washed every 20 min.

The coronary artery rings were first contracted with 60 mmol/L of KCl to determine their maximal contraction tension. After a washout and stabilization period, the rings were contracted with freshly prepared prostaglandin F_2α_ (PGF_2α_; 10^–5^ mol/L in water), a potent coronary vasoconstrictor acting directly on vascular smooth muscle cells, and subsequently relaxed with increasing doses of bradykinin (10^–9^ to 10^–5^ mol/L obtained from a stock solution diluted in 0.1 mol/L acetic acid and conserved at −20°C) to functionally assess the endothelial vasodilatory function. This was repeated (1) after a pre-treatment with indomethacin (10^–5^ mol/L obtained from a stock solution diluted in dimethyl sulfoxide and conserved at −20°C), a cyclo-oxygenase (COX) inhibitor; (2) after a pre-treatment with NG-nitro-L-arginine methyl ester (L-NAME; 2.5 × 10^–4^ mol/L obtained from a stock solution diluted in water and conserved at −20°C), an NO-synthase (NOS) inhibitor; and finally (3) after a pre-treatment with indomethacin (10^–5^ mol/L) and L-NAME (2.5. 10^–4^ mol/L). All drugs were obtained from Sigma–Aldrich Chemicals (Overijse, Belgium). All drug concentrations were expressed as the final concentration in the organ bath. Each experiment set was performed after a washing and stabilization period to allow the vessel rings to return to their basal vascular tone. Contraction to PGF_2α_ was expressed as a percentage of the maximal contraction to KCl, while relaxation to bradykinin was expressed as the percentage of the maximal contraction to PGF_2α_.

### Real-Time Quantitative Polymerase Chain Reaction

Total RNA was extracted from snap-frozen coronary arteries using TRIzol reagent (Invitrogen, Merelbeke, Belgium) followed by a chloroform/ethanol extraction and a final purification using RNeasy^®^ Mini kit (Qiagen, Hilden, Germany), according to manufacturer’s instructions. RNA concentration was determined by spectrophotometry using a Nanodrop^®^ ND-1000 (Isogen life science, De Meern, The Netherlands) and RNA integrity was assessed by visual inspection of GelRed (Biotium, Hayward, CA, United States)-stained agarose gels. Reverse transcription was performed using random hexamer primers and Superscript II Reverse Transcriptase (Invitrogen, Carlsbad, CA, United States), according to manufacturer’s instructions.

For real-time quantitative polymerase chain reaction (RTq-PCR) experiments, sense and anti-sense primers were designed, using Primer3 program, for *sus scrofa* endothelial (eNOS or NOS3), inducible (iNOS or NOS2) and neuronal NOS (nNOS or NOS1), prostaglandin I_2_ receptor (PGI_2_R), prostaglandin F_2α_ receptor (PGF_2α_R), preproendothelin-1 (PPET1), endothelin receptors type A (ET_*A*_) and B (ET_*B*_), thromboxane A2 receptor (TBXA2R), and ribosomal protein L4 (RPL4; used as housekeeping gene) mRNA sequence amplification (Table 1). To avoid inappropriate amplification of residual genomic DNA, intron-spanning primers were selected, when exon sequences were known, and a BLAST analysis was run to check if primer pairs were only matching the sequence of interest. For each sample, amplification reaction was performed in triplicate using SYBR-Green PCR Master Mix (Quanta Biosciences, Gaithersburg, MD, United States), specific primers, and diluted template cDNA. Result analysis was performed using an iCycler system (BioRad Laboratories, Nazareth Eke, Belgium). The analysis of the quantitative RTq-PCR results was done using the change in threshold cycle (△C_t_) value (C_t_ gene of interest − C_t_ reporter gene). Relative gene expression was obtained by △△C_t_ methods (△C_t_ sample − △C_t_ calibrator) using the sham group as a calibrator. The conversion between △△C_t_ and relative gene expression levels was as follows: fold induction = 2^−△△C_t_^ (Winer et al., 1999).

**TABLE 1:** Primers used for real-time quantitative polymerase chain reaction (RTq-PCR) in porcine coronary arteries.

Genes		Sequences
Endothelial nitric oxide synthase (eNOS)	Sense	5′ – CTC TCC TGT TGG CCT GAC CA – 3′
	Antisense	5′ – CCG GTT ACT CAG ACC CAA GG – 3′
Inducible nitric oxide synthase (iNOS)	Sense	5′ – CTG CAT GGA TAA GTA CAG GCT GAC C – 3′
	Antisense	5′ – AGC TTC TGA TCA ATG TCA TGA GCA A – 3′
Neuronal nitric oxide synthase (nNOS)	Sense	5′ – CTT CAA TCT CTT TGT TCA CCT CCT C – 3′
	Antisense	5′ – GAG TAT TAC TCG TCA ATT AAA AGA TTC G – 3′
Prostaglandin I_2_ receptor precursor (PGI_2_R)	Sense	5′ – ATG ATC CGC GGC TTC ACC – 3′
	Antisense	5′ – AAG ACC CAA GGG TCC AGG AT – 3′
Prostaglandin F_2α_ receptor (PGF_2α_R)	Sense	5′ – CAA GGC AGG TCT CAT CAT TTT – 3′
	Antisense	5′ – CAT TGT CAC CAG AAA GGG ACT – 3′
Preproendothelin-1 (PPET-1)	Sense	5′ – TCC TGC TCT TCC CTG ATG GA - 3′
	Antisense	5′ – TGC TCA GGA GTG TTG ACC CA - 3′
Endothelin receptor type A (ETA)	Sense	5′ – TTT ATC CTG GCC ATC CCT CA – 3′
	Antisense	5′ – GCT CTT CGC CCT TGT ATT CAA – 3′
Endothelin receptor type B (ETB)	Sense	5′ – CCC CTT CAT CTC AGC AGG ATT – 3′
	Antisense	5′ – GCA CCA GCA GCA TAA GCA TG – 3′
Thromboxane A2 receptor (TBXA2R)	Sense	5′ – CCT GAA GCC CTG ACC TTT GA – 3′
	Antisense	5′ – CAG ACT CAC CAG CCA GAA GTT – 3′
Ribosomal protein L4 (RPL4)	Sense	5′ – AAA CCA AGG AGG CTG TTC TG – 3′
	Antisense	5′ – CAT TCG CTG AGA GGC ATA AA – 3′

### Statistical Analysis

Normality was tested with a Shapiro–Wilk test. Sphericity was tested with a Mauchly sphericity test. If the sphericity hypothesis was not validated, the Greenhouse–Geisser correction was used. *In vivo* data were analyzed with ANOVA for repeated measurements. *Ex vivo* relaxing concentration–response curves to bradykinin were first assessed using a two-way ANOVA (global curve analysis), followed by Student’s *t*-test for point-by-point analysis when a *p*-value <0.05 was reached. For the relative gene expression, a one-way ANOVA was used. All the tests were performed with a significance level of *p*-value <0.05. All values were expressed as mean ± SEM. All analyses were performed using IBM SPSS Statistics for MacOsX, (SPSS 27.0, IBM, Chicago, IL, United States).

## Results

### General Hemodynamic

In the sepsis group, MAP and LVEDP remained stable along protocol, while HR, CO, and MPAP increased and PaO_2_ / FiO_2_ ratio decreased. General hemodynamic variables remained stable in the sham group, except for a decrease in MAP (Table 2). Left ventricular Ees/Ea (4.5 ± 0.4; 4.3 ± 0.4; 4.1 ± 0.4 vs. 4.1 ± 0.3; 3.4 ± 0.3; 4.3 ± 0.4; *p* > 0.05 respectively at baseline, 6 h, and 12 h for sham and sepsis groups) and right ventricular Ees/Ea were both preserved (2.6 ± 0.4; 1.3 ± 0.2; 1.2 ± 0.4, vs. 3.1 ± 0.8; 2.0 ± 0.5;1.7 ± 0.2; *p* > 0.05 respectively at baseline, 6 h, and 12 h for sham and sepsis groups). Temperature, pH, PaO_2_, PaCO_2_, and hematocrit were maintained constant along the protocol within predefined ranges in both groups.

**TABLE 2:** General hemodynamic and ventilatory data.

Variables	SHAM (*n* = 7)			SEPSIS (*n* = 7)		
	Baseline	6 hr	12 hr	Baseline	6 hr	12 hr
CO (L/min)	4.4 ± 0.4	4.6 ± 0.5	4.2 ± 0.4	4.0 ± 0.4	4.9 ± 0.3*	5.3 ± 0.4^†^
HR (bpm)	80 ± 4	86 ± 7	91 ± 7	86 ± 8	123 ± 7*^§^	126 ± 6^†§^
MAP (mmHg)	88 ± 2	76 ± 2*	73 ± 2^†§^	89 ± 4	85 ± 3	84 ± 3
LVEDP (mmHg)	25 ± 2	26 ± 3	25 ± 4	21 ± 2	25 ± 2	28 ± 3
MPAM (mmHg)	23 ± 1	24 ± 1	26 ± 1	24 ± 1	33 ± 2*^§^	36 ± 2^†‡§^
PaO2/FiO2	416 ± 18	394 ± 21	389 ± 13	386 ± 18	337 ± 24*	293 ± 19^†‡§^

Results are expressed as mean ± SEM. *p < 0.05, 6 h vs. baseline in the same group. ^†^*p* < 0.05, 12 h vs. baseline in the same group. ^‡^*p* < 0.05, 12 h vs. 6 h in the same group. ^§^ *p* < 0.05, sepsis vs. sham group for the same timing. CO, cardiac output; HR, heart rate; MAP, mean aortic pressure; LVEDP, left ventricle end-diastolic pressure; MPAP, mean pulmonary arterial pressure; PaO_2_/FiO_2_, ratio of partial arterial oxygen tension to fraction of inspired oxygen.

### Coronary Pressure–Flow Relationship and Its Autoregulatory Breakpoint

**FIGURE 1:**
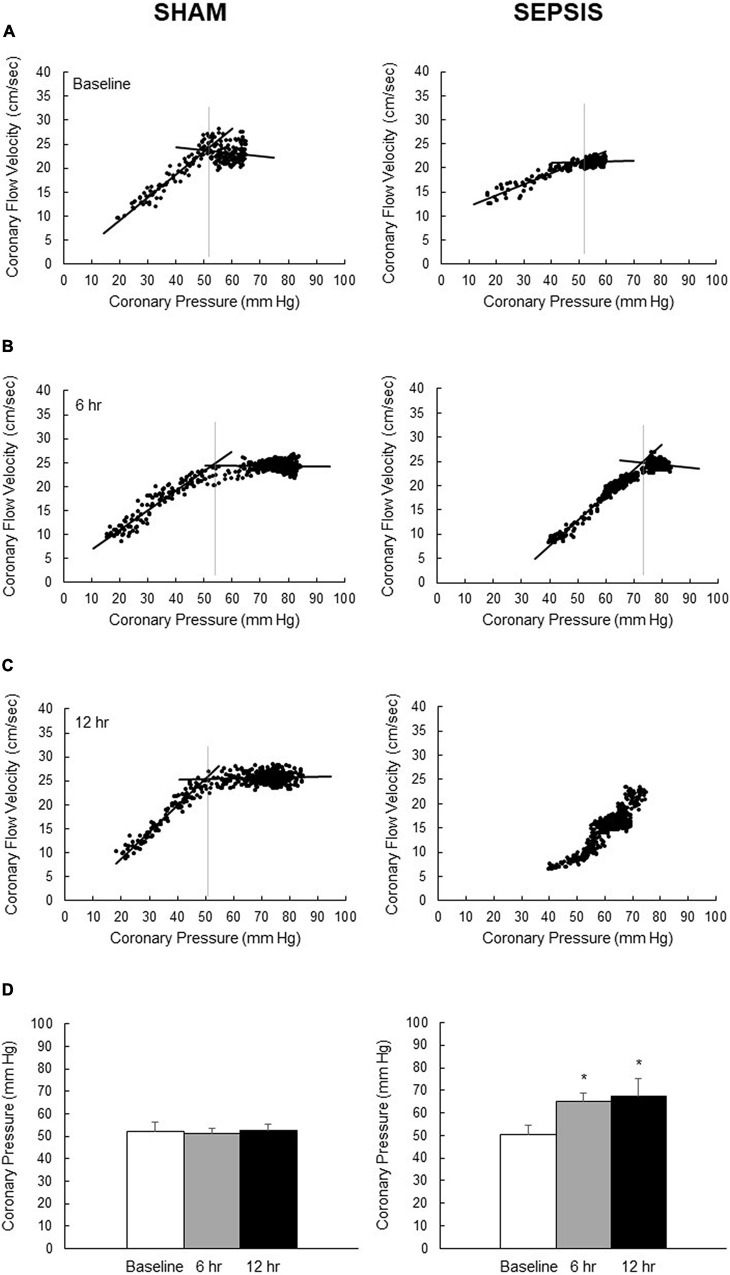
Coronary pressure–flow velocities relationship for the determination of autoregulatory breakpoint pressure. Representative pictures of overtime evolution, beat par beat, of pressure–flow datapoints (black dots) following inflation of the intracoronary balloon to determine the autoregulatory plateau and the ischemic pressure–flow relationship (black lines) and its autoregulatory breakpoint (vertical gray lines) for one representative animal from the sham group (left panel) and one representative animal from the sepsis group (right panel) groups at baseline **(A)**, 6 h **(B)** and 12 h **(C)** after sepsis induction. **(D)** Calculated autoregulatory breakpoint pressure at baseline (whites bars), 6 h (gray bars) and 12 h (black bars) after sepsis induction in sham (left panel; *n* = 4–7) and sepsis (right panel; *n* = 3–7) groups. At 12 h, there were missing data in both groups: the pressure was not sufficiently lowered to calculate the autoregulatory breakpoint for three animals of the sham group; in the sepsis group, the signal was poor for one animal, one pig presented arrhythmia, and two pigs presented a linear pressure–flow relationship between 30 and 75 mmHg with no autoregulatory breakpoint identifiable in this range. Results are expressed as mean ± SEM. *p < 0.01, 6- and 12-h versus baseline in the same group.

At baseline, the lower pressure limit of the coronary pressure–flow relationship was similar in both groups. In Figures 1A–C, which illustrates coronary pressure–flow velocities relationship and autoregulatory breakpoint determination in one representative animal of each group, the autoregulatory breakpoint pressure increased from the 6th h of sepsis, while remaining constant in the sham group (Figure 1D). At 12 h, there were missing data in both groups: the pressure was not sufficiently lowered to calculate the autoregulatory breakpoint for three animals of the sham group; in the sepsis group, the signal was poor for one animal, one pig presented arrhythmia, and two pigs presented a linear pressure–flow relationship between 30 and 75 mmHg with no autoregulatory breakpoint identifiable in this range.

### *In vivo* Coronary Vasomotor Function

#### The Coronary Microcirculatory Dilator Response to Adenosine

**FIGURE 2:**
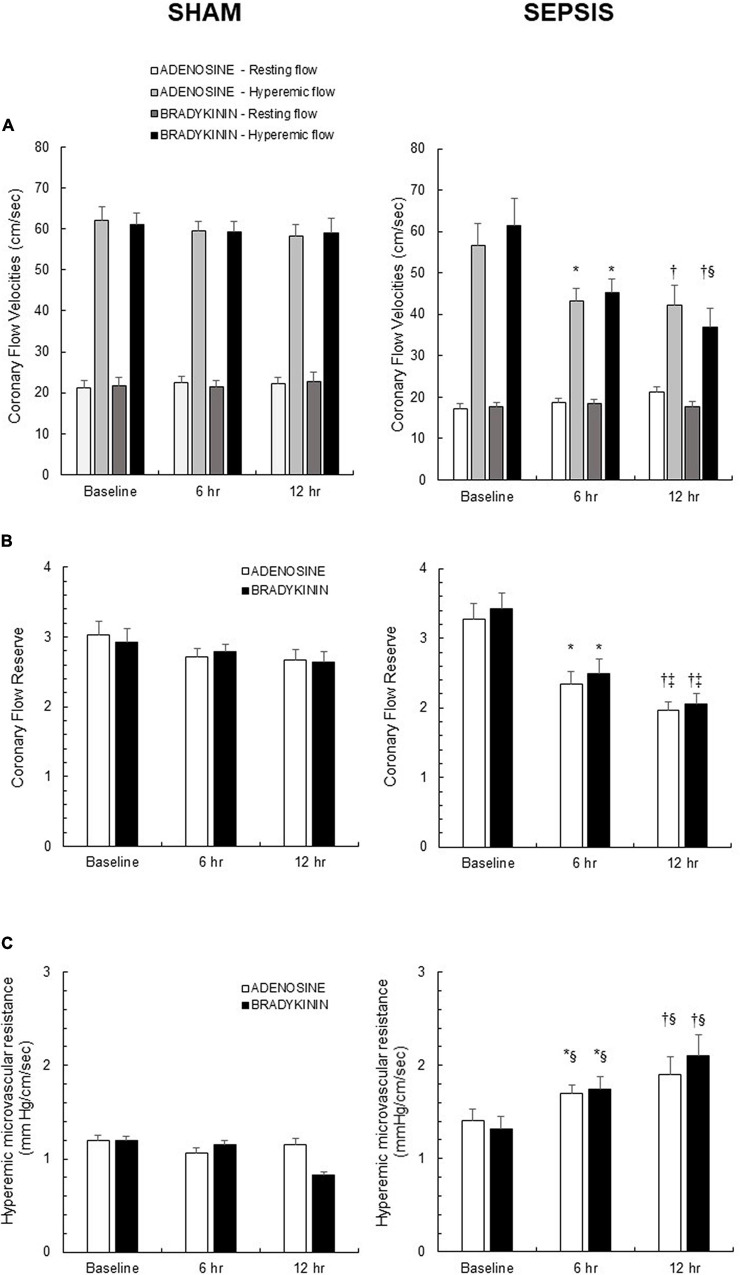
*In vivo* coronary vasomotor response induced by endothelium-independent (adenosine) and endothelium-dependent (bradykinin) vasodilators. **(A)** Resting and hyperemic flows induced by administration of adenosine (white and light gray bars, respectively), and of bradykinin (dark gray and black bars, respectively) in sham (left panel; *n* = 7) and sepsis (right panel; *n* = 7) groups. **(B)** Coronary flow reserve, calculated as the ratio of hyperemic-to-resting flows and representing the metabolic adaptive mechanisms after adenosine (white bars) and bradykinin (black bars) administration in sham (left panel; *n* = 7) and sepsis (right panel; *n* = 7) groups. **(C)** Hyperemic microvascular resistance, calculated as the ratio of coronary pressure-to-hyperemic flow after adenosine (white bars) and bradykinin (black bars) administration in sham (left panel; *n* = 7) and sepsis (right panel; *n* = 7) groups. Results are expressed as mean ± SEM. *p < 0.05 6 h vs. baseline; ^†^*p* < 0.05 12 h vs. baseline; ^‡^*p* < 0.05 12 vs. 6 h after sepsis induction in the same group; ^§^ *p* < 0.05 sepsis vs. sham group for the same timing.

At baseline, both groups were similar with respect to their resting flow and during adenosine-induced hyperemic flow (Figure 2A). All coronary pressure and flow measurements and their derived indices remained stable over time in the sham group. The coronary flow reserve was reduced from the 6th h after the onset of peritonitis (Figure 2B). Similarly, the hyperemic microvascular resistance increased with sepsis development (Figure 2C). These alterations seen in the sepsis group were related to a decrease in hyperemic flow, while the resting flow remained constant (Figure 2A).

#### The Coronary Endothelial Dilator Response to Bradykinin

When coronary pressure and flow measurements were repeated using bradykinin as endothelial-dependent vasodilator, all results were similar to those obtained previously during adenosine-induced vasodilation, indicating a preserved endothelial vasodilatory function in both groups (Figure 2).

### *Ex vivo* Coronary Vasomotor Function

#### The Coronary Contractile Response to PGF_2α_

Maximal contractile responses to PGF_2α_, without pre-treatment, were similar in both sham and sepsis groups (42 ± 7 vs.31 ± 6% of the maximal tension response obtained with 60 mol/L KCl). These maximal contractile responses after indomethacin or L-NAME pre-treatment or after combined indomethacin and L-NAME pre-treatment were respectively 66 ± 6 vs.56 ± 6%; 107 ± 7 vs. 82 ± 4%; and 109 ± 6 vs. 91 ± 4% (*p* > 0.05 for inter-group comparisons).

#### The Coronary Endothelial Dilator Response to Bradykinin

**FIGURE 3:**
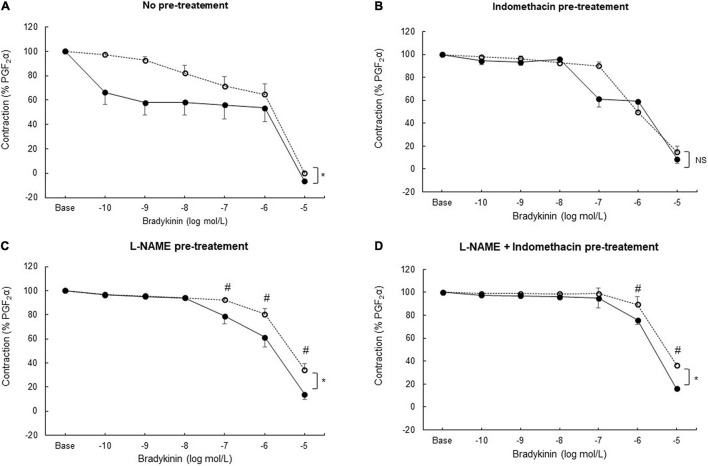
*Ex vivo* coronary vasomotor response. Relaxing concentration–response curves to bradykinin (tested from 10^–10^ to 10^–5^ mol/L) after prostaglandin F_2_α (PGF_2_α; 10^–5^ mol/L) precontraction in coronary arteries rings from sham (open circles; *n* = 25–27 coronary rings from seven pigs) and sepsis groups (solid circles; *n* = 21–24 rings from seven pigs) in the absence of pre-treatment **(A)** and after pre-treatment with a cyclooxygenase inhibitor (indomethacin;10^–5^ mol/L) **(B)**; with a nitric oxide synthase inhibitor (L-NAME; 2.5. 10^–4^ mol/L) **(C)**; with both indomethacin (10^–5^ mol/L) and L-NAME (2.5. 10^–4^ mol/L) pre-treatment **(D)**. Relaxation response was expressed as the percentage of the maximal contraction response to PGF_2α_. Results are presented as mean ± SEM. *p < 0.05 sepsis vs. sham groups (global curve analysis); #*p* < 0.05 sepsis vs. sham groups (point-by-point analysis). NS: not significant.

Bradykinin induced concentration-dependent relaxation in coronary artery rings from both sham and sepsis groups. In the sepsis group, coronary artery rings presented a higher sensitivity to bradykinin (Figure 3A). When inhibiting COX (Figure 3B) or NOS (Figure 3C) respectively by indomethacin or L-NAME pre-treatment, as well as with both pre-treatments (Figure 3D), the maximal relaxation to bradykinin decreased in both groups, compared to the absence of pre-treatment (*p* < 0.05 for all comparisons in the same group). When pre-treated with indomethacin, concentration-dependent vasodilation was similar in both groups. When pre-treated with L-NAME alone or with both L-NAME and indomethacin, the relaxing response to bradykinin was significantly greater in the sepsis group.

### Coronary Artery Expression of Genes Involved in Vasomotion

**FIGURE 4:**
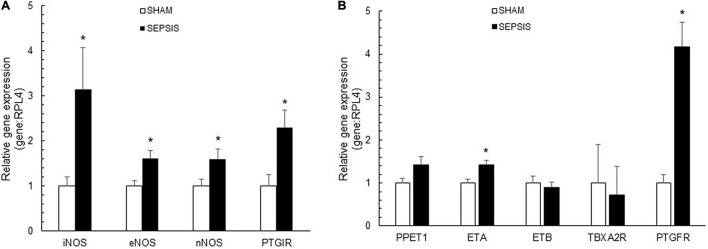
Coronary artery expressions of vasoactive molecules. Relative gene expressions of inducible (iNOS or NOS2), endothelial (eNOS or NOS3), and neuronal (nNOS or NOS1) nitric oxide synthase and prostaglandin I receptor (PTGIR), implicated in vasodilatory pathways **(A)** and relative gene expression of prostaglandin F_2α_ receptor (PTGFR), endothelin receptor type A (ETA) and B (ATB), thromboxane A2 receptor (TBXA2R), implicated in vasoconstrictor pathways **(B)**, in coronary arteries from sham (white bars; *n* = 7) and sepsis (black bars; *n* = 7) groups. Results are expressed as mean ± SEM. *p < 0.05 sepsis vs. sham groups.

In sepsis, coronary arteries presented increased expressions of vasodilatory genes, including iNOS, eNOS, nNOS, and PGI_2_R (Figure 4A). For vasoconstriction-related molecules, septic coronary arteries showed a four-fold increase in relative gene expression of PGF_2α_R compared to the sham group (Figure 4B). Sepsis also induced increased gene expressions of ET_*A*_, while PPET1, ET_*B*_, and TBXA2R gene expressions were similar.

## Discussion

The present results showed that sepsis induced by fecal peritonitis in pigs early altered the coronary blood flow regulation. This coronary blood flow dysregulation was associated with a right shift of the autoregulatory breakpoint pressure, decreased coronary flow reserve, and increased microvascular resistance from the 6th h after sepsis induction, but surprisingly with preserved endothelial vasodilatory response.

Only few previous studies were performed on coronary hemodynamics in sepsis with discordant results. In experimental sepsis induced by endotoxin, coronary blood flow was reduced (Doursout et al., 2000; Soriano et al., 2014; Lorigados et al., 2015), preserved (Breslow et al., 1987; Kang and Kim, 2006), or even restored (with vasopressor treatment) (Lorigados et al., 2015). Increased coronary blood flow was reported in awake animals in hyperdynamic sepsis (Di Giantomasso et al., 2003; Morimatsu et al., 2012). Two studies reported either preserved or increased coronary blood flow (assessed by thermodilution in coronary venous sinus) in human sepsis (Cunnion et al., 1986; Dhainaut et al., 1987). Most patients of these studies benefited from vasopressors and/or inotropic drugs administration, which may be a confounding factor. To our knowledge, the present study is the first which assessed comprehensively the coronary blood flow regulation in a relevant preclinical sepsis model.

**FIGURE 5:**
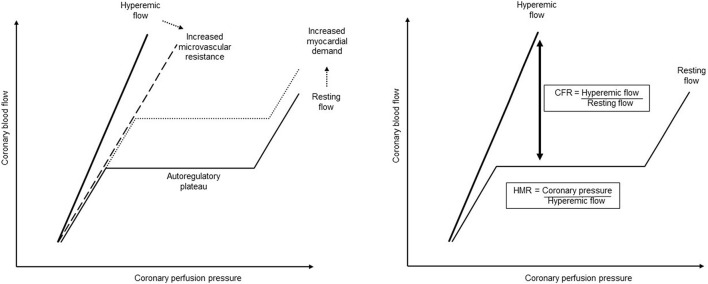
Coronary pressure–flow relationship and its regulation. When autoregulated, the coronary pressure–flow relationship describes a sigmoid curve, where the coronary flow remained constant for a wide range of perfusion pressure, corresponding to the autoregulatory plateau. The autoregulatory breakpoint represents the lower pressure limit below which the autoregulation fails, and the flow becomes directly dependent on the pressure. When the metabolic demand increases, the coronary blood flow increases through the metabolic regulation, corresponding to an upward shift of the autoregulatory plateau. The hyperemic flow represents the coronary blood flow during maximal vasodilation when microvascular resistance is minimal. The slope of this hyperemic flow can be reduced (dashed line) when the microvascular resistance is increased or by endothelial dysfunction. The coronary flow reserve (CFR) represents the remaining possibilities to adapt the coronary blood flow to the myocardial requirement and is so defined as the ration of the hyperemic flow to the resting flow. However, since at maximal vasodilation the coronary flow is directly pressure-dependent, and so also the calculated coronary flow reserve, the hyperemic microvascular resistance (HMR) is proposed as another more reliable pressure–flow derived-index. The hyperemic microvascular resistance represents the microvascular resistance during maximal vasodilation and is defined as the coronary pressure to hyperemic flow.

Sepsis induced a right shift of the pressure–flow relation curve characterized by an increase in the autoregulatory breakpoint pressure, suggesting an increase in microvascular resistance. Sepsis-induced autoregulation impairment has previously been described in the brain (Schramm et al., 2012; Crippa et al., 2018; Ferlini et al., 2020), but never in the coronary circulation. In addition, the resting coronary flow, corresponding to the autoregulatory plateau, remained at its baseline rate, despite the evolution of sepsis. In normal conditions, any increase in heart rate is associated with an upward shift in the autoregulatory plateau of coronary blood flow (Figure 5; Tune et al., 2000; Duncker and Bache, 2008). The cardiac sympathetic autonomic nervous system (ANS) probably plays an important role in this context: it presents a predominant β-adrenergic effect (dilation) on the coronary circulation, while the α-adrenergic effect (constriction) is negligible. During physiological activation of the ANS, resting coronary flow increases proportionately to the resulting increase in the heart rate and inotropism. This modulation of coronary flow is attributed to the direct β-adrenergic vasodilation on the coronary vessels, as well as to local metabolic regulation. However, the tachycardia of the sepsis group was not compensated by an increase in their resting coronary flow, being therefore no longer adapted to the underlying metabolic demand. This observation could be explained by the increased cardiac sympathetic nerve activity described in sepsis (Ramchandra et al., 2009). During increased sympathetic stimulation associated to some pathological conditions, the α-adrenergic coronary constrictor effect increases, to maintain perfusion pressure to the sub-endocardium. Influence of the ANS in coronary flow regulation in sepsis conditions still require further investigation.

Decreased coronary flow reserve observed in septic animals also suggest metabolic regulation impairment. In normal conditions, the coronary blood flow can triple when required, so a coronary flow reserve above 3.0 is considered as normal. Conversely, a coronary flow reserve < 2.0 is considered as pathological and is associated with poor outcome in patients with cardiovascular disease (Kern et al., 2006; Johnson and Gould, 2012; Díez-Delhoyo et al., 2015). In a study concerning 70 patients presenting septic shock with preserved left ventricular ejection fraction, Ikonomidis et al. showed a decreased coronary flow reserve, whose intensity was correlated with the severity of the disease. This coronary flow reserve was a mortality predictor factor, additive to the traditional risk scores, with a threshold of 1.90 (Ikonomidis et al., 2014).

During maximal pharmacological coronary vasodilation, mechanisms controlling microvascular resistance are exhausted and the hyperemic flow becomes directly dependent on the perfusion pressure (Figure 5). A decrease in the slope of this pressure–flow relationship in maximal vasodilation, as observed in the present study, associated with a decreased hyperemic coronary flow, suggested an increase of minimal microvascular resistance (Hoffman, 2000; Johnson and Gould, 2012; Ikonomidis et al., 2014; van de Hoef et al., 2015). As the coronary flow measured during maximal vasodilation was directly correlated to the perfusion pressure, this could introduce a bias to interpret the result. In septic animals, the maintained MAP cannot explain the observed decrease in hyperemic flow. However, to avoid any interference of the coronary pressure on hyperemic flow, and so on the coronary flow reserve, we calculated the hyperemic microvascular resistance index (Figure 5), which reflects the functional state of the coronary microvascular compartment (Kern et al., 2006; Knaapen et al., 2009; Díez-Delhoyo et al., 2015). Septic animals exhibited an increased hyperemic microvascular resistance, which strongly suggested a microvascular impairment. Microvascular dysfunction can be explained by an imbalance between vasoconstrictors and vasodilators. This may concern either an impaired vasodilatory pathway, dependent or not on endothelial function, and/or an inappropriate increase in vasoconstriction. Impaired endothelial vasodilatory response is widely documented in chronic cardiovascular diseases and is related to a loss of vasodilatory pathways, leading to the risk of ischemia when metabolic demand increases (Duncker and Bache, 2008). Here, *in vivo* and *ex vivo* results suggested preserved, even enhanced, endothelium-dependent vasodilatory pathways. These results are consistent with increased gene expression of vasodilatory receptors, NO and prostacyclin, probably interacting together to maintain endothelium-dependent vasodilatation in coronary arteries (Bolz and Pohl, 1997). This is in agreement with the overall increased production of NO in sepsis (Khadour et al., 2002; Trzeciak et al., 2008). Despite preserved endothelium-dependent vasodilatory response, an imbalance in favor of vasoconstrictor influences could explain the observed results in sepsis. In the present study, we showed a significant increase in the receptor of PGF2_α_, which is known to be a potent coronary smooth muscle constrictor (Kromer and Tippins, 1996; Basu, 2007). PGF2_α_ and their metabolites are produced in oxidative stress and inflammation conditions, whether acute or chronic, and seem to play a role in coronary disease (Basu, 2007; Lipcsey et al., 2008). Furthermore, an increased production of PGF2_α_ has already been described in sepsis, especially in the myocardium (Mutschler et al., 2003). Here, we also showed increased expression of PGF receptor in septic coronary arteries. PGF2_α_ could therefore also be involved in the coronary microvascular dysfunction observed in sepsis, and its implication in both peripheral arterial and venous constriction, probably contributing to hyperemic microvascular resistance (Mikkelsen and Andersson, 1978), should be investigated in further studies. Moreover, we cannot exclude that sepsis-induced alteration in PGF2_*a*_ signaling could influence (at least partly) *ex vivo* coronary vasorelaxation testing that was performed after pre-contraction with PGF2_*a*_. It would be therefore interesting to test thromboxane precontraction in further experiments.

The main objective of the present study was to assess the physiology of the coronary circulation and its blood flow regulatory mechanisms during sepsis development. Therefore, we opted for a porcine model of fecal peritonitis-induced sepsis, already recognized and validated for its clinical translational relevance (Kazarian et al., 1994). We also chose to limit the coronary hemodynamic assessment to the early phase of sepsis, in its hyperdynamic state where arterial blood pressure was maintained exclusively by an individualized volume resuscitation, adjusted for hemodynamic parameters without use of vasopressors, to maintain the comparison between the two groups. Indeed, any arterial hypotension, low cardiac output and/or vasopressors use would have induced confounding factors, preventing a reliable interpretation of physiological observations. The septic group benefited from a mixed vascular filling, consisting of both crystalloid and colloid solutions. This could be debated, regarding the adverse effects observed with administration of synthetic colloid solutions in septic shock. Colloids are more efficient volume expanders and persist longer in the vascular compartment, offering the advantage of volume-sparing effect and reduced development of interstitial tissue edema. Moreover, the potential side-effect of colloids may be overlooked in an acute model, as it is in the present study. However, the influence of these different intravascular solutions, including colloids, on coronary blood flow and its regulation has, to our knowledge, never been investigated and could potentially constitute a confounding factor.

The experiments were performed on anesthetized, instrumented, and mechanically ventilated animals, which could dampen a number of reflexes and affect coronary blood flow. This should be therefore considered as a limitation point. To avoid any interference of gender on the response to sepsis development, males and females were equitably distributed between the two groups and such bias were excluded after the analysis of sub-groups. We measured coronary flow velocity and not absolute coronary blood flow, which may be a limitation to our study. However, as the coronary vascular resistance regulation occurs primarily in microcirculation, i.e., distally to our measurement site, we assumed that coronary blood flow and coronary flow velocities were proportional, as discussed elsewhere (Boudart et al., 2020). In addition, unlike methods measuring absolute coronary blood flow, coronary flow velocity measurement is intrinsically corrected for perfused myocardial mass according to the laws of normalized wall shear–stress (Seiler et al., 1993; Johnson and Gould, 2012). The hyperdynamic sepsis was associated to tachycardia, which might also be a limit to the interpretation of our results.

Cardiac dysfunction associated with sepsis is a well-described entity, which is reversible but with a significant morbidity and mortality (Beesley et al., 2018; Sanfilippo et al., 2018). The etiology of the septic cardiopathy is undoubtedly multifactorial, associating mainly direct cardio-depressant effect of circulating substances including NO, prostanoïdes, cytokines, and other inflammatory mediators as well as cellular dysfunction (Antonucci et al., 2014; Beesley et al., 2018). The potential for reduced myocardial blood flow was also mentioned by some (Levy et al., 2005), but remains highly controversial. The present study highlighted coronary blood flow dysregulation associated with microcirculatory dysfunction during hyperdynamic sepsis. This implies that although global coronary blood flow seemed preserved, adequate supply to metabolic demand was lost, reducing the possibilities for further adaptation. Such disturbances could lead to the emergence of hibernating myocardial cells, as proposed by Levy et al. (2005), or even focal ischemia in patients with pre-existing coronary artery disease. Interestingly, alterations of coronary blood flow regulation were not associated with either right or left ventriculo-arterial uncoupling, in spite of already turned-on hyperdynamic state. This observation does not exclude altered coronary function as a contributing factor to altered contractility and ventriculo-arterial uncoupling reported in more advanced stages of the disease (Lambermont et al., 2003).

The results of the present study should guide the physician on the clinical management of septic patients and prompt reflection on arterial blood pressure goals in this context. A deeper understanding of sepsis-induced coronary blood flow dysregulation, more precisely its vasodilatory reserve impairment associated with the shift of its autoregulatory breakpoint toward higher pressures, should encourage personalized blood pressure target depending on the patient (especially in more fragile ones). Pathological conditions, including atherosclerosis, systemic arterial hypertension, and diabetes, are indeed known to alter the mechanisms regulating coronary blood flow, leading to ischemic cardiomyopathy.

In conclusion, this study demonstrates coronary microvascular dysfunction impairing autoregulation and metabolic regulation in a clinically relevant porcine model of hyperdynamic sepsis, but with preserved endothelial vasodilatory response. Manifestations of coronary blood flow dysregulation appear early in sepsis with a right shift of the autoregulatory breakpoint pressure, decreased coronary flow reserve, and increased microvascular resistance from the 6th h after sepsis induction. Such disturbances were not related to changes in mean arterial pressure or cardiac output and could not be avoided by adequate fluid resuscitation, suggesting that the observed coronary blood flow dysregulation occurs even with maintained perfusion pressure.

## Data Availability Statement

The original contributions presented in the study are included in the article/supplementary material, further inquiries can be directed to the corresponding author.

## Ethics Statement

The animal study was reviewed and approved by Comission d’éthique du bien être animal, Faculty of Medecine, Free University of Brussels, Brussels, Belgium.

## Author Contributions

CB, FS, and AH designed the study. CB, FS, LP, AD, PJ, and LD performed the research. CB, PJ, OD, RV, and LD analyzed the data. CB, LD, and RN wrote the manuscript and prepared the figures. SB, JC, RN, LD, and LV helped in the interpretation of data, provided constructive feedback, and revised the manuscript. All authors have approved the final version of the manuscript.

## Conflict of Interest

The authors declare that the research was conducted in the absence of any commercial or financial relationships that could be construed as a potential conflict of interest.

## Publisher’s Note

All claims expressed in this article are solely those of the authors and do not necessarily represent those of their affiliated organizations, or those of the publisher, the editors and the reviewers. Any product that may be evaluated in this article, or claim that may be made by its manufacturer, is not guaranteed or endorsed by the publisher.
